# Pre-surgical Language Mapping in Epilepsy: Using fMRI in Chinese-Speaking Patients

**DOI:** 10.3389/fnhum.2019.00183

**Published:** 2019-06-05

**Authors:** Bing Ni, Xueyuan Wang, Tao Yu, Ruijie Wu, Bo Wang

**Affiliations:** ^1^Department of Functional Neurosurgery, Xuanwu Hospital, Capital Medical University, Beijing, China; ^2^State Key Laboratory of Brain and Cognitive Science, Institute of Biophysics, Chinese Academy of Sciences, Beijing, China; ^3^Beijing MR Center for Brain Research, Beijing, China

**Keywords:** electrocortical stimulation mapping, epilepsy, Chinese language, functional magnetic resonance imaging, language processing

## Abstract

Accurate localization of language processing areas is critical in patients undergoing epilepsy surgery. In this study, we aimed to use functional magnetic resonance imaging (fMRI), which is a non-invasive mapping method, to establish a panel of tasks investigating patients’ language function. We developed six tasks, including a series of progressive comprehension tasks from words, sentence to text, a verb generation task that can detect subtle left-brain activation, an auditory comprehension task that explored the temporal language-related areas, and a visual object-naming task provided for poorly educated patients. We successfully located the language cortex in 40 patients, and subsequently determined hemispheric dominance for the Chinese language. Our results showed a concordance between fMRI tasks and electrical cortical stimulation. The consistency across tasks revealed by the laterality index, as well as the concordance between the surgical outcomes and the results of localization, suggested the validity of our fMRI tasks. Our fMRI tasks also corroborate and extend the finding that the left middle frontal area (BA 9) plays an important role in reading Chinese.

## Introduction

Several approaches have been used to clinically identify the specific cortices and/or dominant hemisphere associated with language, including electrocortical stimulation mapping (ESM) through intracranial electrodes (Ojemann et al., 1989), transcranial magnetic stimulation (Epstein, 1998), the intracarotid sodium amobarbital perfusion test (Wada test) (Strauss and Wada, 1983; Baxendale, 2009), and functional neuroimaging techniques (Bookheimer, 2002).

The Chinese language differs from alphabetical languages such as English in many aspects, including orthography, phonology, and syntax (Kochunov et al., 2003; Qiu et al., 2006). Mandarin has an ideographic script, requiring one to memorize the phonology and meaning of each character to vocalize and comprehend. Thus, brain activation in processing the Chinese language is expected to differ from that in processing English. Valaki et al. (2004) suggested that there was greater symmetry in brain hemispheric dominance in Mandarin speakers evaluated by magnetoencephalography (MEG), whereas asymmetry was obvious in English and Spanish speakers (Valaki et al., 2004). Among those study participants, 100% of the Spanish-speaking population, 80% of the English-speaking population, and only 14% of the Mandarin-speaking population were left-hemisphere dominant.

The purposes of the pre-surgical evaluation for epilepsy include establishing the location and extent of the seizure focus and its relationship to brain function, such as localization of language and other realms of cognitive function. These findings can assist the neurosurgical planning team in determining proper surgical boundaries and preserving vital functions.

In patients with epilepsy, it is especially important to delineate language areas prior to surgery (Duffau et al., 2003; Hamberger, 2007; Cummine et al., 2009), as functional anatomy may be reorganized with a transfer of functions to other areas in the ipsilateral or contralateral hemisphere. For example, functional imaging studies of language processing in patients with chronic epilepsy with a left hemisphere focus have provided evidence for a preoperative right hemispheric activation shift (Billingsley et al., 2001; Gaillard et al., 2002; Rutten et al., 2002; Sabsevitz et al., 2003; Carpentier et al., 2010). Surgical resection of epileptogenic regions requires knowledge about any shift of language function, as it may serve as a prognostic indicator and/or impact the surgical approach, with the potential to minimize neurologic deficits post-surgery.

The ESM is a golden standard for the language mapping. It can directly test any interruption during the stimulation on the language related brain areas. However, for some patients the language task cannot produce any positive result during the stimulation. For the patients whose lesion was on the right hemisphere, the left language related areas could n’t be stimulated due to clinical reason. As a result, fMRI as a non-invasive approach could be an alternative method for those who cannot be tested with ESM.

In our study, we aim to develop a series of fMRI tasks to locate Chinese language related areas before an epileptic surgery. Such tasks should reliably activated Broca’s and Wernicke’s areas on individual level in a relatively short scanning time. We compared our fMRI results with ESM results to verify the validity of our fMRI tasks. Also, we compared the results across different fMRI tasks and calculated the lateralization index to test the reliability of our fMRI tasks.

## Materials and Methods

### Participants

Forty patients (24 male, 16 female) ranging from 9 to 40 years old (mean ± SD: 25.05 ± 9.05 years) were enrolled in the study after giving informed consent. Thirty-six patients were right-handed and four were left-handed native Chinese (Mandarin) speakers from the mainland of China. They all underwent preoperative fMRI between the years 2015 and 2018. In addition, they all met the following inclusion criteria: (1) diagnosed with refractory symptomatic partial epilepsy and sought resective surgery between 2015 and 2018 at the Beijing Institute of Functional Neurosurgery; (2) subjected to non-invasive evaluation of epileptogenic zones in the dominant hemisphere and those potentially close to, or overlapping language areas; and (3) showed signs of normal intelligence and language function preoperatively. The history of epilepsy in these patients ranged from 3 to 38 years (mean ± SD: 13.20 ± 7.47 years). In Table 1, we present whether the resection areas involved Broca’s area (inferior frontal and middle frontal gyri, including Brodmann areas 44, 45, 9, and 46) and Wernicke’s area (supramarginal, angular, and superior temporal gyri, including Brodmann areas 22, 21, 39, and 40). None of the patients’ resection areas included the language cortex defined by our tasks. After surgery, language function in the patients was tested using the Chinese Language Aphasia Test in the immediate postoperative period (3 days post-surgery). The study was conducted in accordance with the ethical principles of the 1964 Declaration of Helsinki. The Institutional Review Boards of the Xuanwu Hospital approved all procedures.

**Table 1:** Descriptive characteristics.

Patients no.	Handedness	Lesion	Surgical resection	Whether BA 9/44/45/46 was involved in resection	Whether BA 21/22/39/40 was involved in resection	Post-surgery language function
1	R	EpiF on LT	LT, LH	No	L BA21, 22	Intact
2	R	LFTP EF	LT, LH, WM D	L BA9, 44, 45, 46	L BA21, 22, 39, 40	Intact
3	R	EpiF on LHT	J of LT, LO, LP	No	L BA39	Intact
4	R	tumor on LT	LT	No	L BA 21, 22	Less fluency
5	R	LF SP	LF	L BA44, 45, 46	No	Intact
6	R	space occupying on L LaV	LT, L LaV	No	L BA21, 22	Intact
7	R	LFTP A	LT	No	L BA21, 22	Intact
8	R	EpiF on LTO	LT, LH	No	L BA21, 22	Intact
9	R	LHemi FCD	F, T, O WM Fiber D	L BA9, 44, 45, 46	L BA21, 22, 39, 40	Intact
10	R	LHemi V, HGM, FCD	LT	No	L BA21, 22	Intact
11	R	LHemi A	F, T, O WM Fiber D	L BA9, 44, 45, 46	L BA21, 22, 39, 40	Intact
12	R	LFIT FCD	LF, LT, LI, LP	L BA9, 44, 45, 46	L BA21, 22, 39, 40	Intact
13	R	EpiF on LF	LT	No	L BA21, 22	Intact
14	R	AO on RF	RT	R BA45, 46	R BA21, 22	Intact
15	R	LHemi A, EF	LT	No	L BA21, 22	Intact
16	R	Cerebellar atrophy	RT, RH, RI	No	R BA21, 22	Intact
17	R	LHemi FCD, LPO EF	J of LP, LO, LT	No	L BA21, 22, 39, 40	Intact
18	R	EpiF on LOT	J of LT, LO, T WM D	No	L BA21, 22, 39, 40	Intact
19	R	No	LT	No	L BA21, 22	Intact
20	R	EpiF on LPF	LP, LF	L BA45, 46	L BA39, 40	Intact
21	R	EpiF on LT	LT	No	L BA21, 22, 39, 40	Intact
22	L	LHemi A, LTP EF	LC, LT, LP, O WM D	No	L BA21, 22, 39, 40	Intact
23	R	EpiF on LO, FCD	J of LT, LO	No	L BA21, 22	Intact
24	R	LFTP EF	LT	No	L BA21, 22, 39, 40	Intact
25	L	J of LFTP EF	LT, LP	No	L BA39, 40	Less fluency
26	R	LFTP FCD	LT, LH, LF, LP,WM D	L BA9, 44, 45, 46	L BA21, 22, 39, 40	Intact
27	R	EpiF on LHT	LT, LP	No	L BA39, 40	Intact
28	R	EpiF on LFT	LF, LP	L BA45, 46	No	Intact
29	R	EpiF on LF	LF, LI	L BA45, 46	No	Intact
30	R	AO on BF, LF HGM	LF	L BA9, 44, 45, 46	No	Less voluntary speaking
31	L	LFTPO EF	Lf, LP	L BA9, 44, 45, 46	L BA39, 40	Intact
32	R	LF FCD	LC, LF	L BA45, 46	No	Less fluency
33	R	EpiF on LT	LT, LP	No	L BA21, 22, 39, 40	Intact
34	L	LH A	LF	L BA9, 44	No	Intact
35	R	LLaV	LF	L BA9, 44, 45, 46	No	Intact
36	R	LH A	LP, LT, LH	No	L BA21, 22, 39, 40	Intact
37	R	EpiF on LT	LT	No	L BA21, 22	Intact
38	R	EpiF on LC	LC, LT, LP	No	L BA39, 40	Intact
39	R	EpiF on LHFT	LF, LT, LP	L BA44, 45, 46	L BA40	Intact
40	R	EpiF on LP	LP	No	No	Intact


*F, frontal; T, temporal; C, Central; P, parietal; O, occipital; R, right; L, left; LaV, lateral ventricle; Hemi, hemisphere; J, junction; WM, white matter; D, disconnection; EF, encephalomalacia focus; EpiF, epileptogenic focus; FCD, focal cortical dysplasia; AO, artery occlusion; HGM, heterotopic gray matter; A, atrophy; SP, schizencephaly; V, ventriculomegaly.*

### Electrocortical Stimulation Mapping (ESM)

Twenty patients underwent craniotomy for implantation of subdural electrodes or stereotactic electroencephalography (sEEG) for localization of epileptogenic foci. Custom subdural grid arrays or sEEG were designed for each patient based on seizure semiology, ictal and interictal scalp video-EEG recordings, and structural MRI.

Intraoperative ESM was performed in patients during the craniotomy. Electrical stimulations were delivered according to the following parameters: bipolar, biphasic, frequency 33 Hz, and pulse duration 0.2 ms, intensity 0.1–18 mA, stimulation duration 3 s. Extraoperative ESM was performed in patients before the craniotomy. Electrical stimulations were delivered according to the following parameters: bipolar, biphasic, frequency 50 Hz, and pulse duration 0.2 ms, intensity 0.1–6 mA, stimulation duration 3 s. The stimulation of language areas was delivered at the threshold previously identified to produce a motor response, which was specific for each patient. During ESM, the intracranial EEG was monitored for after discharges and seizures.

Stimulation was applied to electrodes overlying the areas defined by our fMRI tasks in frontal and temporal lobe language areas. During stimulation, the patients were asked to perform a counting task and a question-and-answer task. If there was a change in language performance during stimulation of a specific electrode, stimulation was subsequently repeated during the testing session to ensure reliability. An anatomical region of the brain was determined as necessary for language function, if errors were observed on testing and repeat testing, and no after discharges or seizures were evident on EEG to account for those errors.

### Language Tasks

Prior to fMRI scanning, tasks were explained to all patients, who were trained in their performance using stimuli different from those presented during the scan, to ensure adequate understanding and performance. In the scanner, all tasks were performed covertly to minimize head motion.

The age and educational background of all 40 patients varied considerably, which led to considerable differences in their ability to complete the language comprehension tasks. We designed three tasks to explore the Broca’s area (inferior frontal and middle frontal gyri, including Brodmann areas 44, 45, 9, and 46).

#### Text Reading

We applied two different texts with about 300 Chinese characters separately in two trials. Each text was shown for 30 s, during which the patient was required to silently read the text and understand its meaning. Three interleaved control trials, each lasting 20 s, were conducted, which entailed the use of a blank screen with only a fixation cross.

#### Sentence Reading

We used a sentence-reading task to test semantic decision making, which robustly activates language-related brain areas, particularly regions contributing to speech perception and lexical-semantic processes. Two test trials (30 s each) were interleaved with three control trials (20 s each). In the test trials, we presented eight simple sentences with a question regarding common sense in daily life that could have been answered with a brief “yes” or “no” (e.g., “Is your name Zhang Xiaohong?”). The patients were required to silently read every sentence and answer each question. All eight sentences were shown on the screen, and the patients were able to adjust their speed of reading. The control trials entailed the use of a blank screen with only a fixation cross.

#### Word Comprehension

We employed a word interpretation task to further investigate activation in Broca’s area. The trial structure was the same as that in the two previous tasks. In the test trials, 16 two- or three-character nouns were shown on a screen. The patients were required to silently read the words and determine whether the object described by the word was living or inanimate. The words in the two test trials were different from each other. In the control trials, a blank screen was shown with a green fixation cross.

Altogether, the three tasks provided a set of complementary tests that could be adapted to various educational backgrounds. Any overlap in the activation areas associated with the three tasks could provide more convincing results regarding language comprehension. In addition to the previous tasks, we also employed a verb generation task, a visual object naming task, and an auditory comprehension task to evaluate more language processes.

#### Verb Generation

Verb generation shows strong left language lateralization indices (LI) in right-handed healthy controls (Petersen et al., 1988). These LIs are consistent with results of more invasive methods, such as Wada testing and intraoperative stimulation in patient populations. In one trial, the patients were instructed to sequentially read 16 nouns, and silently generate a semantically related verb, one after the other (e.g., “dumpling – eat”). Patients were asked to adjust their individual speed of reading. Two 30-s test trials were interleaved with three 20-s control trials. A blank screen with a fixation cross was presented in the control trials.

#### Visual Object Naming

Naming is considered a left-hemisphere function that operates according to a posterior-anterior specificity gradient, with more fine-grained information being processed in the most anterior regions of the temporal lobe (ATL), including the temporal pole (TP). Difficulties in word finding are typically assessed using visual confrontation naming tasks, and have been associated with selective damage to the ATL resulting from various etiologies (Campo et al., 2016). We applied 15 stick figures representing 15 daily objects in each of two test trials. The patients were required to silently name each of the objects in sequence. Each of the two test trials lasted 30 s, which were interleaved with three 20-s control trials. A blank screen with a fixation cross was presented during the control trials.

#### Auditory Comprehension

The auditory comprehension task comprised the auditory version of a word interpretation test. It robustly activated language-related brain areas, particularly those regions contributing to speech perception and lexical-semantic processes. The degree of lateralization of these activated areas corresponds with Wada language asymmetry in individual epilepsy patients. We applied this task to investigate the language processing areas related to Wernicke’s area. The trial structure was the same as those in the previous tasks. In the test trials, the patients passively heard about 10 to 16 words, read in Mandarin, describing daily objects. During a brief pause between the words, the patients were required to silently decide whether the object just referred to was living or inanimate. The number of words heard and length of the brief pause between words varied among patients, based on differences in the educational background of each patient. In the three 20-s control trials, patients only heard the noise of the scanner.

### fMRI Data Acquisition

All imaging was conducted using a three Tesla Siemens Medical systems Prisma scanner at the Beijing Brain MRI Center. Whole brain anatomical scans were acquired using a high resolution magnetization-prepared rapid acquisition gradient echo sequence, consisting of 176 T1-weighted echo-planar image (EPI) slices of 1-mm thickness, with an in-plane resolution of 1 × 1 mm [field of view 256 mm, repetition time (TR) = 2530 ms, echo time (TE) = 3.37 ms]. For each of the functional tasks, T2-weighted single shot gradient echo EPI scans were acquired using an interleaved ascending sequence, consisting of 60 volumes of 25 axial slices of 4-mm thickness with an in-plane resolution of 3.4 × 3.4 mm (field of view = 220 mm, TR = 2000 ms, TE = 30 ms, flip angle = 90°). A 20-channel Siemens head coil was used.

Informed consent complying with local institutional review board regulations was obtained from all patients. An MRI compatible light-emitting diode (LED) projector system was used to show the visual stimulus. The fMRI tasks were all block designed. During each task, a set of two trials (30 s each) was performed and interleaved with three control conditions (20 s each), in which the patient was instructed to maintain fixation.

### fMRI Data Analysis

All preprocessing and statistical analyses for functional images were performed using SPM12 (Statistical Parameter Mapping 12; Wellcome Department of Imaging, London) and the xjView toolbox through MATLAB 8.4 (MathWorks, Inc.). Anatomical and functional scans were reoriented to the anterior commissure, following which the functional images were co-registered to the anatomical scan. Motion correction was implemented using realignment and unwarping. The anatomical scans were segmented into gray matter, white matter, and cerebral spinal fluid, and then normalized to Montreal Neurological Institute (MNI) space. Functional images were transformed into MNI space by using the same normalization parameters. Spatial smoothing was performed using an 8-mm full width at half maximum (FWHM) smoothing function.

For each patient, we modeled fMRI data using the general linear model (spm12). At the individual level, statistical parametrical maps showing brain activation of each language task minus fixation were calculated at the threshold of *p* < 0.001 uncorrected (at most). The usage of an uncorrected statistical threshold is justified because of the clinical reason that we were foremost interested in brain activations at the individual level. Moreover, we believe that the statistical threshold level we chose provides a balance between the risks of type-I versus type-II errors in the statistical analysis of fMRI data, especially when the focus of our test is to investigate brain activity for individual patient. Individual patient’s *p*-value adjustment for each task of activation in language related areas was shown in Supplementary Table S1.

### Lateralization Indices (LI)

Language cortex reorganization was studied in the Broca’s area (inferior frontal and middle frontal gyri, including Brodmann areas 44, 45, 9, and 46) and Wernicke’s area (supramarginal, angular, and superior temporal gyri, including Brodmann areas 22, 21, 39, and 40), using LI to quantify the degree of lateralization of the blood-oxygen-level dependent (BOLD) signal (Rutten et al., 2002; Deng et al., 2015). It was calculated for Broca’s and Wernicke’s areas separately, using the following formula: LI = (VL - VR)/(VL + VR), where VL denotes the number of voxels activated in the left hemisphere and VR denotes the number of voxels activated in the right hemisphere. In Supplementary Table S2, we provided the LIs that were calculated according to Matsuo’s AveLI approach (Matsuo et al., 2012). This result was consistent with previous LIs. The LI ranged from -1 to +1, and language lateralization was categorized into three patterns. An LI less than or equal to -0.2 was considered right-sided lateralization, whereas an LI greater than or equal to 0.2 was regarded as left-sided lateralization, and an LI between -0.2 and 0.2 suggested no clear hemispheric preference (Szaflarski et al., 2002; Backes et al., 2005).

## Results

In the present study, we employed comprehension tasks of text, sentences, visual words, and auditory words for all 40 patients. Moreover, in 29 patients, we added a verb generation task to detect left language lateralization. Among those patients, 20 were given a visual object-naming task in cases of relatively low educational levels. This panel of progressive tasks provided us sufficient information to determine language-related activations in both Broca’s and Wernicke’s areas, as different patients showed different levels of sensitivity to each task. One might elicit weak activation in one task but show strong activation in another. Thus, the overlapping areas of language-related activation in different tasks revealed a more reliable evidence of patients’ language processing areas. Across tasks, we calculated the LI of either overlapping areas in Broca’s area (including Brodmann areas 44, 45, 9, and 46) or overlapping areas in Wernicke’s area (including Brodmann areas 21, 22, 39, and 40). In Table 2, the LI values of the overlapping areas and individual tasks of all patients are presented separately. The lateralization of language processing in each patient was determined according to the majority of LIs within the same indication range. In total, 17 patients showed left-sided lateralization, 18 showed right-sided lateralization, and five showed bilateral processing. When we reported the results of individual patients to the surgeons, we did not emphasize the lateralization of language processing. We aimed to comprehensively demonstrate all brain activations during language processing, especially those adjacent to the ictal focus. Here, we used the concordance between the fMRI and the ESM, LI and its consistency across tasks to measure the validity of the design of our tasks.

**Table 2:** Language lateralization index.

Patients no.	Language lateralization	LI							
		Overlap in Broca’s	Overlap in Wernicke	Text	Sentence	Word	Auditory	Verb generation	Visual object naming
1	bilateral	–0.17	0.75	–0.37	0.50	0.03	0.17	No	No
2	Right	–0.99	–0.90	– 1	– 1	– 1	–0.79	No	No
3	Left	0.46	None	1	0.85	0.33	0.10	No	No
4	Left	0.61	–0.28	–0.08	0.83	0.64	0.59	No	No
5	Right	–1	–0.58	–1	0.02	–0.66	0.13	No	No
6	Left	0.02	1	0.41	0.63	–0.35	–0.04	No	No
7	Right	–0.51	None	–0.22	–0.11	–0.42	–0.95	No	No
8	Right	–0.41	None	–0.35	0.48	–0.48	–1	No	No
9	Right	–1	–1	–1	–0.20	–1	None	No	No
10	right	–0.65	None	None	–1	–0.94	None	No	No
11	right	–0.98	–1	–1	–0.96	–0.28	–1	No	No
12	right	–0.38	None	–0.80	–0.67	None	1	–0.68	No
13	bilateral	0.07	0.01	–0.54	0.09	None	–0.39	0.16	No
14	left	0.01	0.71	0.80	None	0.71	0.12	– 1	No
15	right	–1	–0.49	–1	–1	None	–0.43	–1	No
16	left	0.65	1	0.96	0.58	0.32	–0.14	0.65	No
17	right	–0.58	–0.29	–0.43	None	–0.98	–0.10	–0.79	No
18	right	–0.62	–0.59	–0.96	–0.50	–0.66	–0.40	–0.62	No
19	left	1	0.43	None	1	1	0.33	1	No
20	right	–0.84	– 1	– 1	–0.58	–0.46	–0.02	–0.61	No
21	right	–0.62	None	1	–0.5	–0.45	–0.12	0.19	–1
22	right	–0.45	–0.84	–1	–1	None	–1	–0.54	–1
23	left	0.38	None	0.83	1	0.22	0.68	1	0.74
24	right	–0.55	– 1	– 1	– 1	–0.65	– 1	–0.98	–0.36
25	bilateral	–0.04	–0.02	–0.18	–0.52	–0.35	None	1	–0.55
26	right	–0.85	None	–0.74	0.49	None	–0.57	–0.51	–0.19
27	left	0.24	0.42	0.90	0.97	–0.10	0.22	0.33	None
28	left	0.66	0.25	1	–0.80	None	0.25	0.80	0.92
29	left	0.14	–0.38	0.21	0.69	0.81	–0.92	0.49	0.37
30	left	0.30	–0.33	–0.52	0.22	–0.1	–0.32	0.63	0.50
31	right	–0.89	None	–1	–1	–1	–0.38	–1	–0.81
32	left	0.59	1	0.66	0.79	0.18	None	0.73	None
33	bilateral	–0.13	0.56	–0.30	–0.76	–0.14	0.10	0.78	–0.15
34	left	None	None	None	1	–0.94	None	0.59	None
35	left	0.11	0.68	0.83	0.79	–0.15	0.44	–0.11	None
36	bilateral	–0.60	–0.15	0.93	–0.64	–1	–0.02	None	0.18
37	left	0.71	None	0.55	0.40	0.34	–0.42	0.35	0.53
38	left	0.91	0.08	0.35	None	1	–0.52	0.87	1
39	left	0.54	0.15	–0.97	0.35	0.45	–0.14	0.64	0.29
40	right	–0.18	–0.04	None	–0.77	None	0.07	–0.83	–0.44


*“None” denoted that there was no activation in either Broca’s area or Wernicke’s area during this task when the statistic threshold was set for uncorrected *p* < 0.001, or there was no overlap area across tasks in either Broca’s area or Wernicke’s area when the statistic threshold was set for uncorrected *p* < 0.001. “No” denoted that this patient did not perform this language task.*

### Concordance Between fMRI and ESM

For the 20 patients who underwent ESM, we analyzed both the ESM results and lateralization defined by our fMRI task. Concordance between fMRI and ESM was rated “high” if fMRI identified language in positively mapped sites using ESM, or if fMRI lateralized language to the opposite side of negatively mapped sites (Rodin et al., 2013). Concordance between fMRI and ESM was rated “moderate” if language was found to be lateralized on the right, with some remaining residual activations on the left hemisphere using fMRI, and ESM found positively mapped sites on the left. Concordance between fMRI and ESM was rated “low” if the fMRI failed to detect any activation on the left frontal or temporal lobes, and lateralization was determined to be on the right, and ESM was positive on the left temporal lobe. Results were labeled discordant if fMRI and ESM lateralized language to opposite hemispheres.

Our results (Table 3) showed that 80% of the patients showed high concordance when the fMRI and ESM were compared. Particularly when fMRI lateralized the language function on the left, ESM was able to positively identify mapping on the same side. Moderate concordance was evident in 15% of the patients when comparisons were drawn between the two methods. In these patients, the LIs showed that language lateralization was right-sided, whereas the left hemisphere still showed some activation on the language-related areas, which were identified by ESM. The regions on the right hemisphere were not tested with electrical stimulation, since it was not exposed during the surgery or implanted with electrodes. The anatomical regions identified by ESM and fMRI were not resected during surgery, and post-surgical language function was intact. This suggests that the fMRI findings of left-brain activation were consistent with those of ESM. However, this cannot support our lateralization results on these patients.

**Table 3:** Results of language testing.

Patients no.	Cortical stimulation	Language laterali-zation	Concor-dance rating
8	Positive on left temporal	Right	Low
12	Positive on left frontal and temporal	Right	Moderate
13	Positive on left temporal	Bilateral	High
14	Negative on right frontal and temporal	Left	High
16	Negative on right frontal and temporal	Left	High
17	Negative on left frontal and temporal	Right	High
18	Positive on left frontal and temporal	Right	Moderate
20	Positive on left temporal	Right	Moderate
24	Negative on left frontal and temporal	Right	High
25	Positive on left frontal and temporal	Bilateral	High
26	Negative on left frontal and temporal	Right	High
27	Positive on left frontal and temporal	Left	High
28	Positive on left frontal	Left	High
29	Positive on left frontal and temporal	Left	High
30	Positive on left frontal	Left	High
32	Positive on left frontal	Left	High
33	Positive on left frontal and temporal	Bilateral	High
35	Positive on left frontal and temporal	Left	High
37	Positive on left temporal	Left	High
38	Positive on left temporal	Left	High


Only one patient (5%) showed low concordance between the two methods. The fMRI tasks failed to detect any activation on the left temporal lobe of this patient, whereas ESM produced a positive result on the left temporal lobe. Lateralization, as determined by fMRI tasks, suggested a right-sided language function. However, the post-surgical language function was intact, and part of the temporal lobe was positively mapped by ESM. The reason for the low concordance of this patient is that the ESM result cannot support the right lateralization result. Because this patient was implanted electrodes on the left hemisphere, and resected on the left temporal lobe, there is no chance for us to verify the language function on the right hemisphere with ESM. On the other hand, the intact post-surgical language function maybe depended on the undetected left language related areas or the unverified right language related areas.

### Overlapping Activation Across Tasks

Among the 40 patients enrolled in the study, 39 (97.5%) showed overlapping activation across tasks [29 (72.5%) showed overlapping activations in both Broca’s area and Wernicke’s area; 10 (25%) showed overlapping activations only in Broca’s area]. Of the 39 patients who showed overlapping areas across tasks, 37 (94.9%) showed that the LI of the overlapping areas was consistent with their language lateralization.

Of the 29 patients who performed the verb generation task, only one (3.4%) showed no activation in either left or right language-related areas. In addition, 23 patients (79.3%) showed that the LI of the verb generation task was consistent with language lateralization.

### Language Lateralization

**FIGURE 1:**
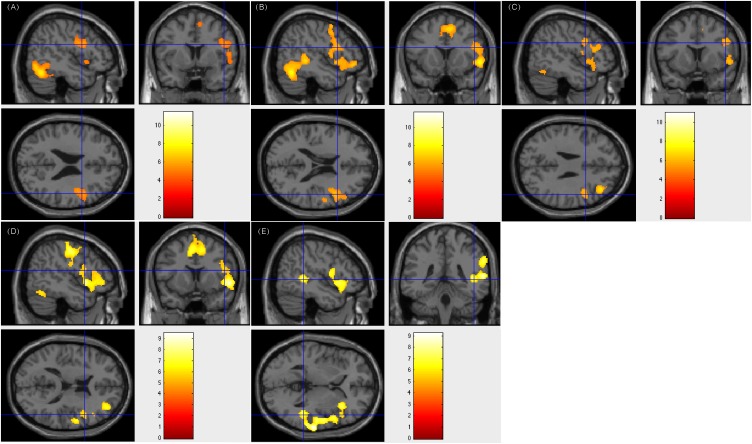
Individual level activation of patient #18 in language related areas showing contrast of task > rest (fdr *p* = 0.001). Different panel showed different task. **(A)** text reading; **(B)** sentence reading; **(C)** word comprehension; **(D)** verb generation; and **(E)** auditory comprehension.

Among the 18 patients showing right-sided lateralization, 17 (94.4%) had resection areas that involved the left Broca’s area and Wernicke’s area. The residual left language area detected by our tasks was remained. Furthermore, these patients all maintained an intact language function after surgery. Current data without ESM confirmed right-sided activation could hardly draw firm conclusion of right dominant language function. However, the fMRI activations across tasks on the right language areas and the intact post-operation language functions provided evidence for the indication of right-sided language lateralization. Of the 5 patients who suffered from multiple left ictal foci in the left hemisphere and underwent white matter disconnection surgery, they all showed right-sided lateralization of language processing. Their post-surgery language function remained intact. Figure 1 showed patient #18’s activation maps of five tasks (A, text reading; B, sentence reading; C, word comprehension; D, verb generation; and E, auditory comprehension).

**FIGURE 2:**
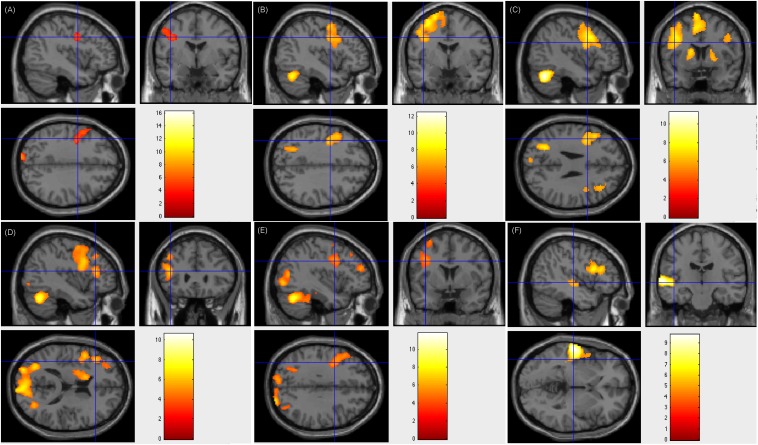
Individual level activation of patient #23 in language related areas showing contrast of task > rest (fdr *p* = 0.001). Different panel showed different task. **(A)** text reading; **(B)** sentence reading; **(C)** word comprehension; **(D)** verb generation; **(E)** visual object naming; and **(F)** auditory comprehension.

Of the 17 patients showing left-sided lateralization, 15 (88.2%) had resection areas that involved the left Broca’s area and Wernicke’s area, and two (11.8%) had resection areas that involved the right Broca’s area and Wernicke’s area. Three of these patients showed less fluency in post-surgical language function, whereas this function remained intact in the rest 14 patients. Figure 2 showed patient #23’s activation maps of five tasks (A, text reading; B, sentence reading; C, word comprehension; D, verb generation; E, visual object naming; and F, auditory comprehension).

**FIGURE 3:**
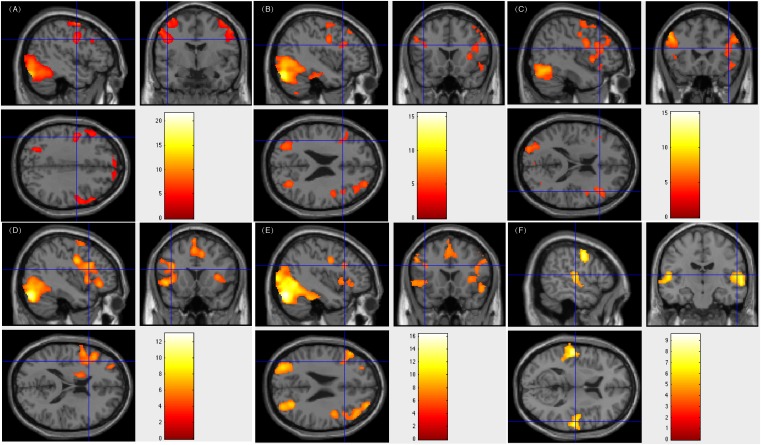
Individual level activation of patient #33 in language related areas showing contrast of task > rest (fdr *p* = 0.001). Different panel showed different task. **(A)** text reading; **(B)** sentence reading; **(C)** word comprehension; **(D)** verb generation; **(E)** visual object naming; and **(F)** auditory comprehension.

All five patients subjected to bilateral language processing had resection areas that involved the left Wernicke’s area. One of these patients showed less fluency in post-surgical language function. Figure 3 showed patient #33’s activation maps of five tasks (A, text reading; B, sentence reading; C, word comprehension; D, verb generation; E, visual object naming; and F, auditory comprehension).

## Discussion

In the present study, we evaluated the efficiency of our fMRI tasks in establishing hemispheric dominance for the Chinese language in patients with epilepsy. Localization of the language cortex and subsequent determination of laterality was successfully achieved in 40 patients. Our fMRI results showed favorable concordance with ESM in patients. We used the LI across various tasks and six individual tasks to measure the laterality for each patient. The relatively high rate of successful laterality assessments, as well as the selection of resection areas according to our assessment guaranteed intact post-surgical language function in patients with epilepsy, and make a strong case for the adoption of our fMRI tasks in Chinese language mapping as a suitable alternative to the Wada test and direct cortical stimulation mapping. The neurosurgeons in our department no longer apply the Wada test because of its invasiveness. Moreover, direct cortical stimulation is also invasive, and as it requires a bone window through which electrodes can be implanted, or an intraoperative wake-up test can be conducted, it may be difficult to perform from a practical perspective (in our study, only half of the patients were able to complete electrical stimulation mapping). Furthermore, direct cortical stimulation was only used for voluntary language assessment on one hemisphere. Our fMRI tasks provided a relatively comprehensive assessment of language-related areas, including both Broca’s area and Wernicke’s area. In order to detect more subtle activations for some of the less activated patients, the control tasks comprised either a blank screen or scanner noise.

The levels of our fMRI tasks, which included reading materials from spoken word to text, were thorough, and progressively investigated the ability to comprehend the Chinese language. The overlapping activation areas across tasks demonstrated the efficiency of the series of tasks among different patients. For clinical purposes, our fMRI tasks could provide more accurate identification of the language-related areas that should be protected during subsequent surgeries.

Our results of the verb generation task revealed that it could easily detect activations of the left hemisphere in language processing. However, it requires better cooperation and higher levels of education. Even in patients with right-sided language lateralization, residual left hemisphere activations were also important for the protection of language function.

Some patients with right-sided language lateralization were performed a white matter disconnection surgery. Limited by the clinical approach, they did not get a chance to verify their fMRI activations on the right language areas by ESM. Reliable fMRI activations on the right language areas helped the surgeons to make a resection plan on the left language areas, and their post-surgical language function remained intact. These results confirm the validity of our approach in assessing the lateralization of language processing.

**FIGURE 4:**
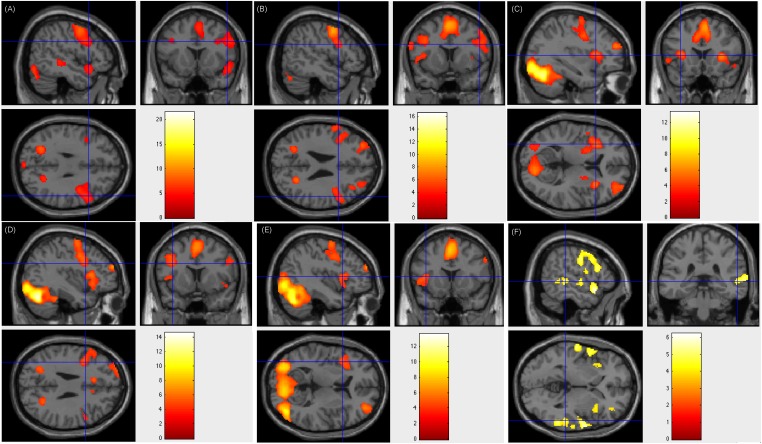
Individual level activation of patient #30 in language related areas showing contrast of task > rest (fdr *p* = 0.001). Different panel showed different task. **(A)** text reading; **(B)** sentence reading; **(C)** word comprehension; **(D)** verb generation; **(E)** visual object naming; and **(F)** auditory comprehension.

Our fMRI tasks also corroborate and extend the finding that the left middle frontal area (BA 9) plays an important role in reading Chinese at multiple levels (text, sentence, word, and verb generation). Previous work (Tan et al., 2000) reported that compared to the fixation baseline, peak activations was elicited in the left middle frontal gyrus (BA 9) when the subjects were required to judge whether a pair of Chinese characters presented synchronously were related semantically. This finding was supported by other studies using similar Chinese word or character retrieval tasks (Tan, 2001a,b; Fu, 2002; Mo et al., 2005; Deng et al., 2015). Using our tasks regarding language comprehension and word retrieval (word generation task), which were different from those of Tan and colleagues, we could locate BA 9 in 39 (97.5%) patients. In the patients with positive results of ESM on left frontal areas, left middle frontal area (BA 9) was verified. However, there were 2 (5%) patients being activated only on left middle frontal area (BA 9), 30 (75%) patients being activated on both left and right middle frontal area (BA 9), and 7 (17.5%) patients being activated only on right middle frontal area (BA 9). Here we show Patient number 30’s activation figures in Figure 4, whose resection area was left frontal area including part of BA 9 and ESM results was overlaid on left BA 9.

Collectively, our findings from multiple tasks in establishing hemispheric dominance for the Chinese language in patients with epilepsy are indicative of the efficiency of this series of fMRI tasks in facilitating surgical planning and the protection of post-surgical language function. Considering the challenges associated with assessing brain function in patients with epilepsy, who have various educational backgrounds, this study supports the utility of this method in pre-surgical language mapping.

The limitation in this method is the need of better design of fMRI tasks, including task control and the balance of scanning time and patient compliance. We intend to add more test blocks and replace the control condition on the premise of good completion. As for patients with various educational backgrounds, it is worth of investigation that whether to develop a method compatible for all the patients or subdivide the test for different groups of patients.

Future studies incorporating a multimodal-imaging framework may further highlight the efficiency of non-invasive methods of functional mapping in challenging populations with epilepsy.

## Ethics Statement

This study was carried out in accordance with the recommendations of “The Institutional Review Boards of the Beijing MR Center for Brain Research” with written informed consent from all subjects. All subjects gave written informed consent in accordance with the Declaration of Helsinki. The protocol was approved by the “Institutional Review Boards of the Xuanwu Hospital.”

## Author Contributions

RW and BN contributed to designing the study and collecting the fMRI data. XW and TY contributed to conducting the ESM and other neurological examinations. RW and BW analyzed the data. BN and RW wrote the manuscript.

## Conflict of Interest Statement

The authors declare that the research was conducted in the absence of any commercial or financial relationships that could be construed as a potential conflict of interest.
