# Supplementary material for: Pre-surgical Language Mapping in Epilepsy: Using fMRI in Chinese-Speaking Patients
Source: Front Hum Neurosci. 2019 Jun 5;13:183. doi: 10.3389/fnhum.2019.00183 (PMC6560162; doi:10.3389/fnhum.2019.00183)
Supplement: Supplementary file 1 [file Table_1.DOCX]

Table S1. Individual patient’s p value adjustment for each task of activation in language areas.

| patients' # | text reading | sentence reading | word comprehension | auditory comprehension | verb generation | visual object naming |
| --- | --- | --- | --- | --- | --- | --- |
| 1 | fwe p=0.05 | fwe p=0.05 | fwe p=0.05 | fwe p=0.05 |  |  |
| 2 | fdr p=0.001 | fdr p=0.001 | fdr p=0.001 | fdr p=0.001 |  |  |
| 3 | fdr p=0.001 | fdr p=0.001 | fdr p=0.001 | fdr p=0.001 |  |  |
| 4 | fdr p=0.001 | fdr p=0.001 | fdr p=0.001 | fdr p=0.001 |  |  |
| 5 | fdr p=0.01 | fwe p=0.05 | fwe p=0.05 | fwe p=0.05 |  |  |
| 6 | fdr p=0.001 | fdr p=0.001 | fdr p=0.001 | fdr p=0.001 |  |  |
| 7 | fdr p=0.001 | fdr p=0.001 | fdr p=0.001 | fdr p=0.001 |  |  |
| 8 | fdr p=0.001 | fdr p=0.001 | fdr p=0.001 | fdr p=0.01 |  |  |
| 9 | fdr p=0.01 | fdr p=0.01 | fdr p=0.01 | fdr p=0.01 |  |  |
| 10 | fdr p=0.01 | fdr p=0.001 | fdr p=0.001 | fdr p=0.001 |  |  |
| 11 | fdr p=0.001 | fdr p=0.001 | fdr p=0.001 | fdr p=0.001 |  |  |
| 12 | fdr p=0.001 | fdr p=0.001 | fdr p=0.001 | fdr p=0.001 | fdr p=0.001 |  |
| 13 | fdr p=0.001 | fdr p=0.001 | fdr p=0.001 | fdr p=0.001 | fdr p=0.0001 |  |
| 14 | fdr p=0.001 | fdr p=0.001 | fdr p=0.001 | fdr p=0.001 | fdr p=0.001 |  |
| 15 | fdr p=0.001 | fdr p=0.001 | fdr p=0.01 | p<0.001 uncorrected | fdr p=0.001 |  |
| 16 | fdr p=0.001 | fdr p=0.001 | fdr p=0.001 | fdr p=0.001 | fdr p=0.001 |  |
| 17 | fdr p=0.001 | fdr p=0.01 | fdr p=0.001 | fdr p=0.001 | fdr p=0.001 |  |
| 18 | fdr p=0.001 | fwe p=0.05 | fwe p=0.05 | fwe p=0.05 | fwe p=0.05 |  |
| 19 | fdr p=0.001 | fdr p=0.001 | fdr p=0.01 | fdr p=0.01 | fdr p=0.001 |  |
| 20 | fdr p=0.01 | fdr p=0.001 | fdr p=0.001 | fdr p=0.001 | fdr p=0.001 |  |
| 21 | fdr p=0.001 | fdr p=0.01 | fdr p=0.001 | fdr p=0.001 | fdr p=0.001 | fdr p=0.001 |
| 22 | fdr p=0.01 | fdr p=0.01 | fdr p=0.05 | fdr p=0.001 | fdr p=0.01 | fdr p=0.01 |
| 23 | fdr p=0.001 | fdr p=0.0001 | fwe p=0.05 | fwe p=0.05 | fwe p=0.05 | fwe p=0.05 |
| 24 | fdr p=0.001 | fdr p=0.0001 | fdr p=0.001 | fdr p=0.0001 | fdr p=0.001 | fdr p=0.001 |
| 25 | fdr p=0.001 | fdr p=0.001 | fdr p=0.01 | fdr p=0.01 | fdr p=0.01 | fdr p=0.01 |
| 26 | fdr p=0.01 | fdr p=0.001 | fdr p=0.001 | fdr p=0.001 | fdr p=0.001 | fwe p=0.05 |
| 27 | fdr p=0.001 | fdr p=0.001 | fdr p=0.001 | fdr p=0.001 | fdr p=0.001 |  |
| 28 | p<0.001 uncorrected | p<0.001 uncorrected | p<0.001 uncorrected | p<0.001 uncorrected | p<0.001 uncorrected |  |
| 29 | fdr p=0.001 | fdr p=0.001 | fdr p=0.001 | fdr p=0.001 | fdr p=0.001 | fwe p=0.05 |
| 30 | fwe p=0.05 | fwe p=0.05 | fwe p=0.05 | fwe p=0.05 | fwe p=0.05 | fdr p=0.001 |
| 31 | fdr p=0.01 | fdr p=0.001 | fdr p=0.001 | fdr p=0.001 | fdr p=0.001 | fdr p=0.001 |
| 32 | fdr p=0.001 | fdr p=0.001 | fdr p=0.001 | fdr p=0.001 | fdr p=0.001 | fdr p=0.001 |
| 33 | fwe p=0.05 | fwe p=0.05 | fwe p=0.05 | fwe p=0.05 | fwe p=0.05 | fdr p=0.001 |
| 34 | no activation | p<0.001 uncorrected | p<0.001 uncorrected | p<0.001 uncorrected | p<0.001 uncorrected | p<0.001 uncorrected |
| 35 | fdr p=0.001 | fdr p=0.001 | fdr p=0.001 | fdr p=0.001 | fdr p=0.001 | fdr p=0.001 |
| 36 | fdr p=0.001 | fdr p=0.001 | fdr p=0.001 | fdr p=0.001 | fdr p=0.001 | fdr p=0.001 |
| 37 | fdr p=0.001 | fdr p=0.001 | fdr p=0.001 | fdr p=0.001 | fdr p=0.001 | fdr p=0.001 |
| 38 | p<0.001 uncorrected | p<0.001 uncorrected | p<0.001 uncorrected | p<0.001 uncorrected | p<0.001 uncorrected | p<0.001 uncorrected |
| 39 | fdr p=0.001 | fdr p=0.001 | fdr p=0.001 | fdr p=0.001 | fdr p=0.001 | fdr p=0.001 |
| 40 | p<0.001 uncorrected | p<0.001 uncorrected | p<0.001 uncorrected | p<0.001 uncorrected | p<0.001 uncorrected | p<0.001 uncorrected |

Table S2. Individual AveLI of each task.

| Patients  No. | Language  lateralization | LI | | | | | | | |
| --- | --- | --- | --- | --- | --- | --- | --- | --- | --- |
|  |  | Overlap in Broca’s | Overlap in Wernicke | Text | Sentence | Word | Auditory | Verb  generation | Visual object naming |
| 1 | bilateral | -0.12 | 0.54 | -0.27 | 0.4 | 0.12 | 0.15 | no | no |
| 2 | right | -0.89 | -0.8 | -1 | -1 | -1 | -0.68 | no | no |
| 3 | left | 0.38 | none | 1 | 0.76 | 0.41 | 0.15 | no | no |
| 4 | left | 0.7 | -0.3 | -0.12 | 0.73 | 0.6 | 0.52 | no | no |
| 5 | right | -1 | -0.47 | -1 | 0.1 | -0.54 | 0.07 | no | no |
| 6 | left | -0.15 | 1 | 0.25 | 0.45 | -0.28 | -0.15 | no | no |
| 7 | right | -0.39 | none | -0.25 | -0.18 | -0.38 | -0.87 | no | no |
| 8 | right | -0.28 | none | -0.4 | 0.32 | -0.37 | -1 | no | no |
| 9 | right | -1 | -1 | -1 | -0.1 | -1 | none | no | no |
| 10 | right | -0.53 | none | none | -1 | -0.75 | none | no | no |
| 11 | right | -0.79 | -1 | -1 | -0.87 | -0.12 | -1 | no | no |
| 12 | right | -0.42 | none | -0.68 | -0.52 | none | 1 | -0.49 | no |
| 13 | bilateral | 0.15 | 0.11 | -0.42 | -0.12 | none | -0.25 | 0.09 | no |
| 14 | left | -0.05 | 0.65 | 0.69 | none | 0.62 | 0.02 | -1 | no |
| 15 | right | -1 | -0.39 | -1 | -1 | none | -0.35 | -1 | no |
| 16 | left | 0.58 | 1 | 0.78 | -0.47 | 0.28 | -0.2 | 0.46 | no |
| 17 | right | -0.45 | -0.2 | -0.34 | none | -0.79 | -0.25 | -0.79 | no |
| 18 | right | -0.52 | -0.41 | -0.85 | -0.35 | -0.46 | -0.28 | -0.52 | no |
| 19 | left | 1 | 0.35 | none | 1 | 1 | 0.23 | 1 | no |
| 20 | right | -0.67 | -1 | -1 | -0.42 | -0.35 | -0.12 | 0.06 | -1 |
| 21 | right | -0.48 | none | 1 | -0.32 | -0.39 | -0.02 | 0.05 | -1 |
| 22 | right | -0.38 | -0.69 | -1 | -1 | none | -1 | -0.38 | -1 |
| 23 | left | 0.25 | none | 0.68 | 1 | 0.08 | 0.52 | 1 | 0.56 |
| 24 | right | -0.38 | -1 | -1 | -1 | -0.48 | -1 | -0.86 | -0.2 |
| 25 | bilateral | -0.1 | 0.12 | -0.08 | -0.42 | -0.16 | none | 1 | -0.38 |
| 26 | right | -0.76 | none | -0.62 | 0.29 | none | -0.39 | -0.41 | -0.22 |
| 27 | left | 0.34 | 0.4 | 0.87 | 0.87 | -0.02 | 0.3 | 0.35 | none |
| 28 | left | 0.54 | 0.15 | 1 | -0.6 | none | 0.28 | 0.75 | 0.78 |
| 29 | left | 0.02 | -0.3 | 0.15 | 0.58 | 0.72 | -0.85 | 0.37 | 0.28 |
| 30 | left | 0.25 | -0.18 | -0.35 | 0.28 | -0.02 | -0.24 | 0.58 | 0.39 |
| 31 | right | -0.68 | none | -1 | -1 | -1 | -0.26 | -1 | -0.74 |
| 32 | left | 0.39 | 1 | 0.56 | 0.68 | 0.05 | none | 0.58 | none |
| 33 | bilateral | -0.1 | 0.48 | -0.22 | -0.68 | -0.02 | 0.15 | 0.69 | -0.08 |
| 34 | left | none | none | none | 1 | -0.83 | none | 0.48 | none |
| 35 | left | 0.02 | 0.52 | 0.7 | 0.62 | -0.02 | 0.34 | -0.03 | none |
| 36 | bilateral | -0.48 | -0.05 | 0.78 | -0.49 | -1 | -0.1 | none | 0.1 |
| 37 | left | 0.65 | none | 0.35 | 0.26 | 0.24 | -0.32 | 0.28 | 0.35 |
| 38 | left | 0.75 | 0.02 | 0.27 | none | 1 | -0.43 | 0.78 | 1 |
| 39 | left | 0.46 | 0.1 | -0.79 | 0.33 | 0.34 | -0.02 | 0.47 | 0.27 |
| 40 | right | -0.08 | -0.14 | none | -0.59 | none | 0.02 | -0.67 | -0.32 |
